# Different Dietary Approaches, Non-Alcoholic Fatty Liver Disease and Cardiovascular Disease: A Literature Review

**DOI:** 10.3390/nu15061483

**Published:** 2023-03-20

**Authors:** Jose D. Torres-Peña, Antonio P. Arenas-de Larriva, Juan F. Alcala-Diaz, Jose Lopez-Miranda, Javier Delgado-Lista

**Affiliations:** 1 Lipids and Atherosclerosis Unit, Department of Internal Medicine, Maimonides Biomedical Research Institute of Cordoba (IMIBIC), Reina Sofia University Hospital, University of Cordoba, Av. Menéndez Pidal s/n, 14004 Cordoba, Spain; 2 CIBER Fisiopatología de la Obesidad y Nutrición (CIBEROBN), Instituto de Salud Carlos III, 28029 Madrid, Spain

**Keywords:** non-alcoholic fatty liver disease, Mediterranean diet, intermittent fasting, low-fat diet, ketogenic diet, low-carbohydrate diet, DASH diet, cardiovascular disease

## Abstract

Non-alcoholic fatty liver disease (NAFLD) is the first cause of chronic liver disease and is also associated with other harmful entities such as obesity, metabolic syndrome, dyslipidemia, and diabetes. NAFLD is a significant public health concern worldwide, impacting individuals of all ages, and its prevalence is projected to increase in the near future due to its connection with obesity. Intrinsic (genetics) and external (lifestyle) factors may also modulate NAFLD, and, in turn, may partly explain the observed relationship between NAFLD and cardiovascular disease (CVD). Although many drugs are been tested to treat NAFLD, to date, no drug has indication to specifically treat this disorder. Thus, the current management of NAFLD relies on lifestyle modifications and specifically on weight loss, physical activity, and the intake of a healthy diet. In the present narrative review, we will discuss the effects of certain dietary patterns on NAFLD incidence and progression.

## 1. Introduction

Non-alcoholic fatty liver disease (NAFLD) is the first cause of chronic liver disease. The range of progression may vary from the mildest stage, non-alcoholic steatohepatitis to fatty liver, fibrosis, and the most severe, such as cirrhosis and even hepatocellular carcinoma [1], and it is well described that this condition is associated with obesity, dyslipidemia, and diabetes [2]. Insulin resistance, impaired lipid metabolism, inflammation, and the presence of oxidative stress disrupt hepatocyte homeostasis and ultimately, lead to their death. After hepatocyte death, the process of repairing an injury begins when signals are released, activating immune cells and sinusoidal endothelium, astrocytes, and ductal cells, leading to fibrogenesis and endothelial remodeling of the affected area [3].

It is important to highlight that there are inherited factors such as genetic polymorphisms in the codifying DNA of some enzymes or proteins associated with hepatic steatosis, cancer, and hepatocytes lipids content, have an crucial role in NAFLD [4,5]. The specific nutrigenomic effects of diets on these genes are beyond the scope of the present review but constitute an interesting line of research for future studies. 

Although this review will focus mainly on diet, other environmental-related factors may modulate NAFLD. An example of this is the pieces of evidence on the bidirectional effects of lifestyle on the gut–liver axis. Environmental factors of any subject (diet, sleep, or feeding habit, among others) influence the gut microbiota, which, in turn, affects the body weight or the hepatic health of the subject [6,7,8].

### 1.1. The Complex Nexus between Cardiovascular Disease and NAFLD. NAFLD Pharmacotherapy and Dietetic Approach to Managing NAFLD

The link between NAFLD and CVD is complex, involving various environmental factors such as sedentary lifestyles, diets high in saturated fats, sleep disorders, increased visceral fat, and obesity. Although certain genetic polymorphisms are linked to NAFLD, most are also associated with triglycerides, cholesterol concentrations, and atherosclerosis [7]. NAFLD is characterized by high levels of antagonists of NO synthase [8] and a procoagulant state [9], which increases the risk of CVD. Both NAFLD and atherosclerotic disease share risk factors such as changes in glucose metabolism or insulin resistance [10].

Lifestyle modifications such as weight loss and physical activity are key management strategies for NAFLD [11]; however, pharmacotherapies are being evaluated as well [12]. These include statins, metformin [13], thiazolidinediones, omega-3 [14], and novel therapies [15,16,17,18] such as Acetyl-CoA inhibitors, Peroxisome Proliferator-Activated-Receptor agonists, Glucagon-Like Peptide 1 Agonists, Caspase inhibitors, and Sodium-Glucose Cotransporter-2 Inhibitors

This review will examine relevant literature on nutritional approaches for managing NAFLD.

### 1.2. Clinical Practice Guidelines for the Dietary Treatment of NAFLD

The Clinical Practice Guidelines of the American Association of Clinical Endocrinology state that the primary treatment of NAFLD is weight loss with a low-calorie diet, restriction of saturated fat, starch, and carbohydrates, improved eating patterns, and exercise. Cardiometabolic benefits and reduction of liver fat can be observed with >5% weight loss. More weight loss provides increased benefits and may reverse steatohepatitis or even liver fibrosis (10% weight loss) [19]. Regarding specific dietary patterns, several societies including the European Association for the Study of the Liver, the European Association for the Study of Diabetes, the European Association for the Study of Obesity [20], the European Society for Clinical Nutrition and Metabolism [21], the Asian Pacific Association for the Study of the Liver [22], and the American Gastroenterological Association [23] specifically recommend the Mediterranean diet for persons with NAFLD. Some of these guidelines [20,23] also include recommendations not only regarding the type of pattern, but also provide advice on macronutrient composition, i.e., restriction of simple carbohydrates and high glycemic carbohydrates or restriction of total and saturated fat and increase in monounsaturated fatty acids (MUFAs) and omega 3 polyunsaturated fatty acids (n-3 PUFAs). These recommendations are based on the evidence that support that regardless of weight loss, these macronutrients improve metabolic parameters, decrease the liver enzymes levels and reduce steatosis in NAFLD further than weight loss alone [24].

## 2. Nutritional Treatment in NAFLD

**Figure 1:**
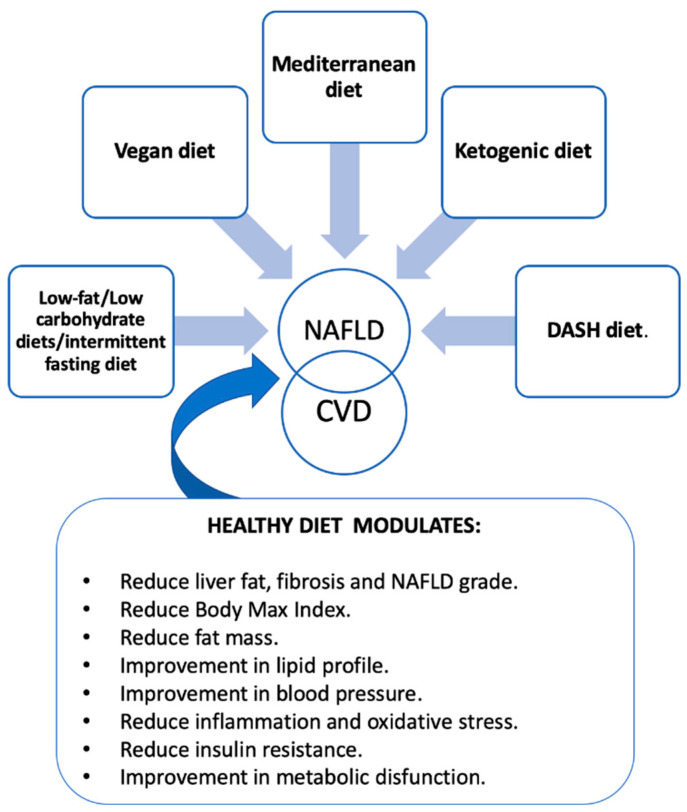
NAFLD, diet and CVD. NAFLD: non-alcoholic fatty liver disease; DASH: Dietary Approaches to Stop Hypertension; CVD: cardiovascular disease.

Healthy dietary patterns characteristics and their potential effects on NAFLD (see Figure 1).

As we previously mentioned, the pathogenesis of NAFLD is related to many interactions between environmental and genetic factors [4,5]. An important point is insulin resistance, which plays a key role in the appearance of NAFLD and its progression to different stages of chronic liver disease [10]. It is well known that insulin resistance displays an increased hepatic lipogenesis, a reduced inhibition of lipolysis in the adipose tissue, and an increased fatty acids transport to the liver, resulting in adipose tissue dysfunction and disrupted production of cytokines [10]. These metabolic changes are characterized by elevated concentrations of free fatty acids and promote lipotoxicity, with an increase in oxidative stress. As we mentioned above, another factor that influences the pathogenesis of NAFLD is the gut microbiota, which may modify free fatty acids absorption and modulate the production of inflammatory cytokines [6,7,8]. In this section, we will review how different dietary patterns may focus on these two main pathways involved in NAFLD and may exert a beneficial role in liver steatosis (see Table 1).

### 2.1. Mediterranean Diet and NAFLD

Characterized by a high proportion of fruits, vegetables, legumes, cereals, white meat, and fish, and with olive oil as the main source of fat, the Mediterranean diet is recognized as a healthy diet around the world. This diet has a minimum of 35% of the calories as fats, around 15% proteins, and a maximum of 50% carbohydrates.

A large body of research supports the beneficial role of a Mediterranean diet in chronic diseases [25,26,27,28,29,30,31,32]. Is important to highlight two studies here that support the benefits of the Mediterranean diet in the prevention of cardiovascular disease. The effectiveness of the Mediterranean diet in primary prevention of cardiovascular disease was demonstrated in the PREDIMED study [25] when compared to a control diet. Furthermore, the CORDIOPREV study [26], conducted by our group, assessed the long-term effects of a high-intensity dietary intervention based on either a Mediterranean or low-fat diet in 1002 patients with coronary heart disease. The study showed that the Mediterranean diet was superior to the low-fat diet in preventing new major cardiovascular events.

Besides these cardiovascular benefits, in recent years, there is a growing interest in establishing the relationship between the consumption of a Mediterranean diet and NAFLD. In this context, observational studies and clinical investigation trials support the concept that the Mediterranean diet may prevent NAFLD. First, in an observational study, adherence to the Mediterranean diet was associated with lower insulin resistance and liver steatosis in patients with NAFLD [33]. In the cross-sectional analysis of two independent studies, the UK Fenland Study and the Swiss CoLaus Study (almost 14,000 participants), a higher adherence to the Mediterranean diet was associated with a lower prevalence of hepatic steatosis assessed by ultrasound [34]. Another observational study conducted on more than 500 individuals with cardio-metabolic risk factors, including obesity, showed an inverse correlation between NAFLD and the Mediterranean diet. Additionally, a higher adherence to the Mediterranean Diet was associated with lower insulin resistance among these patients [35]. In this line, Kouvari et al. [36] evaluated in the ATTICA prospective cohort study the association between the Mediterranean diet, hepatic steatosis, and fibrosis in patients with NAFLD, with and without diabetes, obesity, and established cardiovascular disease. They reported that the Mediterranean diet protected against diabetes and CVD among subjects with NAFLD and additionally showed an inverse association between the adherence to the Mediterranean diet and NAFLD. Secondly, several trials evaluated the effect of the Mediterranean Diet on NAFLD. The method of evaluation of liver steatosis in these trials varies and includes, among others, liver ultrasound and magnetic resonance. In a first prospective study [37], fifty overweight patients with body mass index greater than 25 kg/m^2^ were randomized into three groups. A low-calorie Mediterranean diet was prescribed to Group A and B patients for six months. In association with the diet, Group B patients were administered two pills of antioxidant pills. Patients of the control group (C) were advised to reduce body weight. The investigators showed that participants in the two Mediterranean diet groups improved their anthropometric parameters (all *p* = 0.001), and lipid profile (*p* = 0.020) and reduced their hepatic fat (*p* = 0.017), compared to the other group. In addition, antioxidant supplementation improved insulin sensitivity (*p* = 0.045). In another study [38], investigators evaluated the effect of a low glycemic index Mediterranean diet on the NAFLD score as measured by liver ultrasonography in 98 participants. The Mediterranean diet was found to decrease the NAFLD score after six months [OR: 0.07 (CI: 0.02–0.12; *p* < 0.05)]. In contrast to the previous study, although both excluded patients without known cardiovascular disease and the intervention period was six months, the majority of patients had overweight or obesity (97%), presented with moderate NAFLD, and were also patients without diabetes.

Other studies with a duration of less than or equal to 3 months have also evaluated the effect of this diet model on NAFLD.” Specifically, Properzi et al. [39] performed a trial to evaluate the effect of two isocaloric diets (Mediterranean or low fat) on liver steatosis over 3 months measured with magnetic resonance). In this case, both diets, low-fat and Mediterranean diets, improved liver steatosis compared to baseline (*p* < 0.01) but without difference between both dietary patters (*p* > 0.05).In a report from Ryan et al. [40], twelve non-diabetic subjects with biopsy-proven NAFLD were recruited for a randomized, cross-over 6-week dietary intervention study. All subjects took both the Mediterranean and low fat-high carbohydrate diet in random order with a 6-week wash-out period. The Mediterranean diet, in contrast to the previous study, reduced liver steatosis and improves insulin sensitivity, compared to the low fat-high carbohydrate diet (39 ±4% vs. 7 ± 3%; *p* = 0.012).

Data on reviews and meta-analyses agrees with the above-reviewed evidence. In a recent meta-analysis [41], investigators showed that the available data to date suggest that there is an inverse relationship between Mediterranean diet intake and liver steatosis [0.95 (CI: 0.9–1); *p* = 0.05], probably with the implication of improvements in some factors such as body mass index, (Effect size = −1.23 kg/m^2^ (CI: −2.38 to −0.09), triglycerides (Effect size = −33.01 mg/dL (CI: −52.84 to −13.18), or insulin resistance/insulin sensitivity (Effect size = −0.94; CI: −1.29 to −0.58). In a second meta-analysis, Haigh et al. [42] reviewed data from randomized and clinically controlled trials describing the effects of the Mediterranean diet and calorie restriction on NAFLD biomarkers. The meta-analysis showed that dietary interventions reduce alanine aminotransferase (*p* < 0.001), aspartate aminotransferase (*p* = 0.004), fatty liver index (*p* < 0.001), and liver steatosis (*p* = 0.02). They concluded that data suggest that the Mediterranean diet may be an effective diet when treating NAFLD. A third interesting meta-analysis [43] investigated the role of a Mediterranean diet on liver steatosis and insulin resistance in patients with NAFLD. In that report, the Mediterranean diet was associated with reduced fatty liver index (CI: −0956 to −0.237; *p* = 0.001) and lower HOMA-IR (CI: −0.713 to −0.003; *p* = 0.048) when compared with the control diets. If we evaluate the population characteristics of these meta-analyses, we found that the meta-analysis of Haigh et al. included 54.7% male, mean age of 50 years, and BMI > 31; thus, these “population baseline characteristics” were similar to those reported in the other two meta-analysis.

To sum up, the previous data support the beneficial role of the Mediterranean on NAFLD. In this context, observational studies and clinical investigation trials support the concept that the Mediterranean diet may prevent NAFLD by a direct influence of this dietary pattern in some metabolic factors such as insulin sensitivity/resistance, lipids metabolisms, and body max index. Thus, the Mediterranean diet may act as a beneficial nutritional approach in patients with NAFLD.

In line with the above, several current reports of European and American Scientific societies on the management of NAFLD identify the Mediterranean diet as the first choice for the prevention/treatment of NAFLD and its complications [20,21,22,23].

### 2.2. Low-Fat Diet and NAFLD

The low-fat diet restricts the amount of energy obtained from fat sources. Typically, a low-fat diet limits energy from fat to no more than 30% of total daily calories [44]. Low-fat diets have been investigated extensively to evaluate their efficacy in body weight loss and the associated beneficial changes [45,46]. Additionally, low-fat plans have been shown to reduce risk factors of metabolic syndrome [47]. However, it is unclear if this dietary strategy is also successful in treating NAFLD. In one trial in participants with NAFLD, hepatic triglyceride content fell by 25% after 12 weeks of low-fat diet (*p* < 0.01), independently of body weight loss and or caloric intake [39]. In another study of an 18-month weight-loss trial in 139 participants with abdominal obesity/dyslipidemia (which compared low-fat vs. Mediterranenan/low-carbohydrate diet), a low-fat diet resulted in a reduction in hepatic fat content of 3.8% (*p* < 0.001) [48]. Additionally, in this study, the authors showed that decreases in the proportion of liver fat content seem to act as a stronger mediator of the favorable effects of the low-fat diet on some cardiometabolic markers than does general visceral fat reduction. Finally, it is important to highlight, as complementary information of the previous section (Mediterranean Diet and NAFLD), that the authors reported that the reduction in liver fat content was greater in the MedDiet/Low-Carbohydrate group (*p* < 0.05).

Despite these findings, recent studies suggest that a low-carbohydrate diet may be more appropriate than low-fat diets for the treatment of NAFLD. The available evidence of this fact will be reviewed in the Section 2.5.

### 2.3. The Dietary Approaches to Stopping Hypertension (DASH) and NAFLD

The DASH diet has been used as a lifestyle approach for treating and preventing hypertension [49,50]. It consists of a diet low in saturated fats, high in proteins, fiber, minerals and low in sodium [49]. The DASH eating plan is based on fruits, vegetables, low-fat dairy products, fish, whole grains, poultry, nuts, seeds, and legumes, while reducing the consumption of fat, red meat, and products with added sugar [50]. The DASH diet has demonstrated to reduce mortality from all causes, CVD, diabetes, and cancer [51].

Recently, the relationship between NAFLD and the DASH diet has attracted attention, due to the evidence brought from some observational studies and one clinical trial, which may support that the prevention of NAFLD could be achieved through the DASH dietary pattern.

One study [52] showed an inverse relationship between adherence to the DASH diet and NAFLD risk. Specifically, participants in the highest quartile of the DASH Diet Score had a thirty percent reduction in the risk of NAFLD (OR: 0.70; 95% CI: 0.61, 0.80). In line with these results, a report from a long-term study evaluating a large cohort after 2 decades of follow-up showed that being in the highest tertile of adherence to the DASH diet was associated with a lower risk of NAFLD (OR between 0.57–0.77) [53].

Another two studies showed the benefits of DASH on NAFLD: on the one hand, the Multiethnic Cohort [54] reported an inverse association between DASH scores and NAFLD risk (OR: 0.78; CI: 0.69–0.89); and, on the other hand, a subgroup from the HELENA trial showed that DASH scores were inversely associated with liver fat content (OR: 4.41; *p* = 0.05) [55]. Finally, in a recent report, the DASH diet was proposed as an effective tool for the management of NAFLD [56].

The evidence from clinical trials is limited. Only a clinical trial on 60 overweight and obese patients with NAFLD randomly assigned participants to either the DASH diet or a control diet for eight weeks. In this trial, the consumption of the DASH diet had beneficial effects on weight, body max index (*p* = 0.06), liver enzymes (*p* < 0.05), triglycerides (*p* = 0.04), insulin resistance (*p* = 0.01), and inflammatory markers (*p* < 0.05) [57].

In summary, evidence from some observational studies and one clinical trial may support that DASH dietary patterns could be a preventive tool for NAFLD. However, more clinical trials are needed to further evaluate the findings from a heterogeneous population of the observational studies.

### 2.4. Vegetarian Diets and NAFLD

The accumulated research on the effects of vegetarian diets on liver biochemistry and histology in NAFLD patients is limited. The available evidence comes from some cross-sectional and short-term clinical trials. Even after adjusting for gender, age, smoking, and alcohol consumption, among others, a cross-sectional study [58] involving 1273 vegetarians and 2127 non-vegetarians revealed that vegetarians had a significantly lower risk of developing NAFLD than non-vegetarians (OR: 0.79; *p* < 0.05). In addition, compared to vegetarians, non-vegetarians had a higher fibrosis score. The authors hypothesized that the liver benefits seen with this diet could be attributed to abundant polyphenol content of the vegetarian diet, which can reduce insulin resistance, oxidative stress, and inflammation. Another study [59] showed that this diet was linked to a reduction in fasting glucose, insulin resistance, body max index, cholesterol (total and LDL-C) (all *p* < 0.05), and a 57% reduction in the new onset of fatty liver disease [OR: 0.43 (CI: 0.32–0.87); *p* = 0.013]. Finally, a three-month randomized clinical trial [60] in seventy-five overweight/obese patients with NAFLD evaluated a ovo-lacto-vegetarian diet followed by standard weight loss. Fifty-four patients on a lacto-ovo-vegetarian diet had significant improvements in body max index, waist circumference, liver enzymes, fasting blood glucose, insulin resistance, lipid profile, systolic blood pressure, and NAFLD showed significant improvement compared to people on a weight loss diet (67% vs. 21%; *p* = 0.01). 

To sum up, all these findings highlight the need for research to evaluate the specific impact of the vegetarian diets on liver histology in NAFLD patients.

### 2.5. Low/Very Low-Carbohydrate Ketogenic Diet and NAFLD

A recent meta-analysis [61] found that neither low-carbohydrate/very low carbohydrate are better in improving liver fat or transaminase levels in NAFLD compared to a low-fat diet. Additionally, there is evidence that beyond the distribution of macronutrients in the diet, the type of calories may be considered. Concerning carbohydrates, the glycemic index, related to the postprandial glycemic response, may be a useful tool in the management of NAFLD. In this context, there are different studies [62,63] that have incorporated a low glycemic index diet into their nutritional interventions with a positive effect on the liver fat composition. In obese people, a ketogenic low-carbohydrate diet was shown to drastically lower hepatic triglycerides concentrations [64]. In addition to promoting weight reduction, these diets may also have positive effects on the liver disease by reducing insulin levels, lipogenesis, and fatty acid oxidation [65]. Thus, in a 12 weeks randomized, controlled trial with obese participants with the polycystic ovarian syndrome, the ketogenic diet was superior to a control diet in lowering liver enzymes and improving the fat liver content by liver ultrasound, thus the ketogenic diet improved menstrual cycle, body weight, blood glucose and liver function test (all *p* < 0.05) at 12 weeks. The ketogenic diet group reduced liver function test compared to control group (*p* < 0.05) [66]. Although these results are very interesting, it is difficult to extrapolate them to other populations due to the specific pathogenic mechanisms involved in the poly-cystic ovarian syndrome. In another trial, additionally, obese patients after a 2-month intervention with a ketogenic diet reduced body weight (−9.7 kg vs. −1.67 kg; *p* < 0.0001), visceral, and hepatic fat more effectively than a traditional low-calorie diet (4.77 vs. 0.79; *p* < 0.005) [67]. Swift weight loss and fast mobilization of liver fat may offer an effective alternative for NAFLD treatment, as suggested by these findings.

### 2.6. Intermittent Fasting and NAFLD

This dietary strategy produces an energy reduction by restricting the feeding period. Intermittent fasting is a term used to describe various methods of energy restriction, which range from alternating periods of eating and fasting to complete abstinence from food or consumption of very low energy [68].

Periodic fasting showed to reduce NAFLD in 697 participants in a prospective observational trial in participants with or without type 2 diabetes [69]. After intermittent fasting, there was a decrease in fasting glucose, glycated hemoglobin, body mass index, and liver enzymes. In addition, the number of fasting days was positively correlated with an improvement in the fatty liver index. Moreover, at the end of the study, 50 per cent of the subjects with baseline fatty liver index over 60 (which is the lower limit for high FLI, and confirms NAFLD) changed to intermediate or low FLI, suggesting liver disease regression. In another controlled trial, seventy patients with NAFLD were assigned to an intermittent calorie restriction diet, low-carbohydrate diet, or general lifestyle advice for 3 months. Participants in the intermittent calorie restriction diet reduced body weight and liver steatosis compared to the general lifestyle advice (−2.6%; CI: −5.0 to −0.2) [70]. It is also important to note that, in this study, the low-carbohydrate diet (these data support the evidence reported in the previous section of these manuscript) was associated with a reduction in hepatic fat content (−3.9; CI: −6.3 to −1.4).

Two relevant trials [71,72] evaluated alternated-day fasting and established an association of these intermitted fasting methods with a reduction in liver fat and fibrosis score. Finally, a recent meta-analysis, with 417 NAFLD participants from six trials, showed the positive effects of intermittent fasting on liver tests [73]. 

In summary, intermittent fasting includes different methods of energy restriction. In this intermittent fasting, different strategies may have a potential positive role in patients with NAFLD. However, further studies are necessary to confirm the beneficial impact of variations of intermittent fasting on the onset and evolution of NAFLD.

**Table 1:** Summary of the main characteristics of trials investigating the effects of healthy diet on NAFLD. Abbreviations: NAFLD: non-alcoholic fatty liver disease; MRI: Magnetic Resonance; T2DM: type 2 diabetes; MedDiet: Mediterranean diet; IR: insulin resistance; CVD: cardiovascular disease; ALT: alanine aminotransferase; AST: aspartate aminotransferase.

Author [Ref.]	Study Design	Population Health Status	Sample Size	Duration	Type of Intervention/Diet Evaluated	Type of Control	Main Findings
Kontogianni et al. [33]	Observational	Recent NAFLD diagnosis	73	-	MedDiet	-	MedDiet negatively correlated with liver enzymes (*p* = 0.03), insulin resistance (*p* = 0.001) and severity of steatosis (*p* = 0.006).
Khalatbari-Soltani et al. [34]	Cross-sectional	Two adult populations (Fenland and CoLaus cohorts)	13.602	-	MedDiet	-	Greater adherence to MedDiet was associated with lower liver steatosis [0.86 (CI: 0.81–0.90)].
Baratta et al. [35]	Observational	Healthy adults	584	-	MedDiet	-	Higher adherence to MedDiet was associated with lover IR (OR: 0.801; *p* = 0.018) and NAFLD (high adherence vs. low OR: 0.093; *p* = 0.030).
Kouvari et al. [36]	Observational	NAFLD	3032	-	MedDiet		Higher MedDiet score was inversely associated with NAFLD [0.53 CI: 0.29–0.95)].
Abenavoli et al. [37]	Randomized trial	Patients with overweight and NAFLD	50	6 months	A.MedDiet B.MedDiet+ antioxidant.	Standard of care diet	MedDiet alone or with antioxidant improves insulin sensivity (*p* = 0.045), HOMAR-IR (*p* = 0.021), Tryglicerides index (*p* = 0.020) and fatty-liver index (*p* = 0.017) and anthropometric parameters (all *p* = 0.001).
Misciagna et al. [38]	Randomized controlled trial	Moderate to severe NAFLD	98	6 months	Low glycemic index Mediterranean diet	Standard of care diet	Low glycemic index Mediterranean diet decreases NAFLD score [OR: 0.07 (CI: 0.02–0.12; *p* < 0.05)]
Properzi et al. [39]	Randomized controlled trial	NAFLD with cardiometabolic risk factors	56	12 weeks	MedDiet	Low-fat diet	Both diets improve liver steatosis (*p* < 0.01) without difference betweens both groups (*p* = 0.32)
Ryan et al. [40]	Randomized controlled trial	NAFLD patients with obesity	12	6 weeks	MedDiet	Low fat-high carbohydrate diet	MedDiet reduces liver steatosis (39 ± 4% vs. 7 ± 3%; *p* = 0.012).
Akhlaghi et al. [41]	Meta-analysis	NAFLD patients	17.095	-	MedDiet	-	A trend for the improvement of NAFLD was observed for MedDiet [0.95 (CI: 0.9–1); *p* = 0.05].
Kawaguchi et al. [43]	Meta-analysis	NAFLD patients	250	-	MedDiet	Standard of care diet	MedDiet improved liver steatosis (CI: −0956 to −0.237; *p* = 0.001) and IR (CI: −0.713 to −0.003; *p* = 0.048).
Haigh et al. [42]	Meta-analysis	NAFLD patients	3037	-	MedDiet with calorie restriction	Standard of care diet	MedDiet reduced ALT (*p* < 0.001), AST (*p* = 0.004), fatty liver index (*p* < 0.001) and liver steatosis (*p* = 0.02).
Gepner et al. [48]	Randomized controlled trial	Patients with obesity/dyslipidemia	278	-	Low fat	MedDiet/Low-carbohydrate	Liver fat content reduction in all groups (- 4%compared to baseline, *p* < 0.001). Greater in the MedDiet/Low-carbohydrate group (*p* = 0.036) was observed.
Hekmatdoost et al. [52]	Case-control	NAFLD patients	306	-	DASH diet	Standard of care	Inverse relationship between the DASH diet and NAFLD risk (OR: 0.70; *p* < 0.05).
Park et al. [54]	Observational	Multiethnic Cohort	2959	-	DASH diet	DASH diet highest vs. lower quartile	High DASH scores are associated with a reduction in NAFLD risk (OR: 0.78; *p* < 0.001).
Watzinger et al. [55]	Cross-sectional	NAFLD patients	136	-	DASH diet and MedDiet	-	Diet quality scores was inversely associated with liver fat content (OR: 4.41; *p* = 0.04 for MedDiet score and OR: 4.41; *p* = 0.05 for DASH score).
Razavi Zade et al. [57]	Randomized controlled trial	Overweigh and obese patients with NAFLD	60		DASH diet	Standard of care	DASH diet reduced body max index (*p* = 0.06), liver enzymes (*p* < 0.05), triglycerides (*p* = 0.04), insulin resistance (*p* = 0.01) and inflammatory markers (*p* < 0.05).
Chiu et al. [58]	Cross-sectional	Non-vegetarians and vegetarians	3400	-	Vegetarian diets	-	Vegetarian diets may be inversely associated with NAFLD (OR: 0.79; *p* < 0.05).
Jin et al. [59]	Observational	Vegetarians	339	-	Vegetarian diet		Vegetarian diet, was associated with lower odds of NAFLD [OR: 0.43 (CI: 0.32–0.87); *p* = 0.013] and cardiometabolic risk factors(Body max index, LDL cholesterol, fasting glucose and insulin resistance, all *p* < 0.05).
Garousi et al. [60]	Randomized controlled trial	Overweight/obese adults with NAFLD	75	3 months	Lacto-ovo-vegetarian diet.	Standard weight-loss diet	Lacto-ovo-vegetarian diet reduced grade of NAFLD compared to Standard weight-loss diet (67% vs. 21%; *p* = 0.01)
Ramon-Krauel et al. [62]	Randomized controlled trial	Obese children	17	6 months	Low-glycemic load	Low-fat diet	Both diets improved liver steatosis (without differences between the two diets)
Ahn et al. [61]	Meta-analysis	Heterogeneus population with NAFLD	370	-	Low-carbohidrate diet	Low-fat diet	No differences between Low-carbohidrate and Low-fat diet
Li et al. [66]	Randomized controlled trial	Polycystic ovary syndrome	20	12 weeks	Ketogenic diet	Conventional pharmacological treatment	Ketogenic diet improved menstrual cycle, body weight, blood glucose and liver function test (all *p* < 0.05) at 12 weeks.Ketogenic diet group reduced liver function test compared to control group (*p* < 0.05).
Cunha et al. [67]	Randomized controlled trial	Healthy participants	39	2 months	Very low-calorie ketogenic diet	Low-calorie diet	Very low-calorie ketogenic diet reduced weigh (−9.7 kg vs−1.67 kg; *p* < 0.0001) and liver fat (4.77 vs. 0.79; *p* < 0.005)compared to low-calorie diet.
Drinda et al. [69]	Observational	T2DM and non-T2DM participants	697	-	Periodic fasting	-	Periodic fasting with weigh reduction rapid improved fatty liver index (Non-T2DM participants: −14.02 compared to baseline; *p* < 0.0001 and T2DM participants −19.15; *p* < 0.001 compared to baseline. Additionally, greater changes in T2DM participants *p* < 0.002 T2DM vs. non-T2DM).
Cai et al. [71]	Randomized controlled trial	NAFLD patients	271	12 weeks.	Alternate-day fasting	Standard of care	Changes in fat free mass, lipids, fasting insulin, blood pressure and liver stiffness in both groups compared to baseline without differences between the two groups.
Holmer et al. [70]	Randomized controlled trial	NAFLD patients	74	12	Interminent calorie restriction and low-carbohydrate high-fat diet	Standard of care	Intermintent calorie restriction diet reduced hepatic fat content (−2.6%; CI: −5 to −0.2). Low-carbohidrate high fat diet reduce hepatic fat (−3.9%; CI: −6.3 to −1.4)
Yin et al. [73]	Meta-analysis	NAFLD patients	417	-	Intermittent fasting	-	Intermittent fasting improved weigh (−2.45%, CI: −3.98 to −0.91; *p* < 0.05) and liver enzymes (ALT: −10.54, CI: −14.01 to −7.08; *p* < 0.05 and ALT: −11.31, CI: −14.3 to −8.32; *p* < 0.05).

## 3. Factors That Modulate Dietary Patterns

The way people eat can vary greatly depending on their lifestyle and culture [74]. Factors such as health problems, physical changes, economic status, and social circumstances may make it difficult to choose a healthy and varied diet that meets nutritional needs [75].

First, a person’s socioeconomic status is a significant factor in determining their eating habits [76]. This status can be determined by various factors such as education level, financial stability, and personal perception of social standing. In this context, research indicates that countries with higher incomes tend to have a positive correlation with a higher socioeconomic status, which, in turn, leads to healthier food choices and a higher consumption of fruits and vegetables [76]. In addition, low socioeconomic status groups consume less dairy products, more saturated fats, carbohydrates, and foods with more calories than high groups [77]. The main reason for this phenomenon is that high-fat and high-carbohydrate foods tend to have a lower price and are more easily accessed than other, healthier foods, and these foods give the consumer “low cost” energy.

Moreover, age and marital status influence eating habits; there is a strong correlation between being unmarried and an increased risk of dying from cardiovascular disease [78]. This is due to the changes in eating habits that are caused by social isolation, which results in a reduction in the consumption of fruits and vegetables and an increase in the consumption of fats and carbohydrates. This fact is amplified in older people.

Finally, nutrition knowledge is another key point that influence dietary patters. Thus, some reports show that an approach to address obesity in adults through nutritional education is associated to weight loss, changes in eating patterns, and a decrease in fat intake [79].

To sum up, the factors that modulate dietary patterns include socioeconomic status, with higher income groups tending to have healthier food choices; access to and affordability of food, with cheaper, high-fat, and high-carbohydrate foods being more easily accessible; age and marital status, with unmarried and older individuals at increased risk of poor dietary habits; and nutrition knowledge, with education and awareness leading to positive changes in eating patterns.

## 4. Future Perspectives

Available human studies have shown the beneficial effects of different dietary patterns in NAFLD. However, more randomized controlled trials are required to verify these results, in some of the diets used. In this context, several clinical trials are currently ongoing to evaluate the beneficial effects of dietary interventions in patients with NAFLD. Although this review is not a systematic review or a metanalysis, to evaluate the trials involving diet and NAFLD, we searched the ClinicalTrial.gov and EudraCT databases and searched relevant trials using the topic research fields “NAFLD” and “Diet,” selecting those trials with active recruiting (see Table 2). The types of diets to be evaluated in these studies are very varied and include the Mediterranean diet (NCT05275608 and EudraCT:2021-000152-19), low-calorie diets (NCT05268042), ketogenic diets (NCT05275608, NCT04383951), fat-restricted diets (NCT05268042), and even a combination of a Mediterranean diet with different antidiabetic drugs (EudraCT:2021-000152-19). Additionally, the target population of these trials include overweight and obese patients, patients with prediabetes, and T2DM patients.

## 5. Take Home Message for Healthcare Givers

Based on all the evidence we have evaluated throughout this review, we would like to highlight some dietary recommendations from a practical point of view that may be very useful for healthcare givers:

1. Reduce total energy intake: moderate calorie restriction (500–1000 kcal/day that can be achieved with different dietary pattern such us intermittent fasting, low carbohydrate diets, etc.) can improve NAFLD. Patients with NAFLD should aim to lose 5–10% of their body weight over a period of six months.
2. Follow a Mediterranean-style diet or DASH diet: a Mediterranean diet is rich in fruits, vegetables, whole grains, legumes, nuts, fish, and olive oil, low in red and processed meat, refined carbohydrates, and saturated fat. This dietary pattern is associated with a lower risk of NAFLD and its complications.
3. Limit added sugars and refined carbohydrates: high intake of added sugars and refined carbohydrates increases the risk of NAFLD. Patients should avoid or limit intake of sugar-sweetened beverages, sweets, and high-calorie snacks.
4. Avoid alcohol consumption: Alcohol is a hepatotoxin and can worsen liver damage in patients with NAFLD. Patients with NAFLD should avoid alcohol or limit consumption to no more than one drink per day for women and two drinks per day for men.

## 6. Final Remarks

In this review, we summarized the most relevant available evidence and updated information about NAFLD. We reviewed new knowledge on the effectiveness of different nutritional therapies in managing NAFLD. We highlight the importance of weight loss in patients with overweight or obesity, and note how pharmacological therapy can be combined with diet changes in advanced cases of NAFLD. Additionally, we emphasize the importance of maintaining long-term adherence to a personalized diet, and, to our knowledge, that some other types of diets besides the Mediterranean diet, such as low-fat, vegetarian, and intermittent diets, could be effective alternatives for treating NAFLD, according to evidence from observational and prospective studies.

NAFLD is a significant public health concern worldwide, impacting individuals of all ages, and its prevalence is projected to increase in the near future due to its connection with obesity, diabetes mellitus, metabolic syndrome, cirrhosis and liver cancer or cardiovascular disease. In this review, we have described different nutritional therapies valid, effective or that have shown promising results in NAFLD management. Thanks to the positive effects found with the different healthy diets, clinicians may prescribe individualized healthy diets depending on the individual preferences of each patient and the particular effect searched. In all cases, weight loss when overweight or obesity are present shall be a priority. Additionally, pharmacological therapy in combination with diet may be one cornerstone when treating NAFLD not responding to dietary intervention alone, or in advanced stages of NAFLD. Additionally noteworthy of consideration is the fact that one of the main issues regarding diet as part of the treatment of NAFLD is the maintainance of long-term (possibly lifelong) adherence. Thus, clinicians and nutritionists should explore different dietary patterns with their patients and establish a personalized dietary plan that considers each patient’s preferences, the accumulated evidence on each diet benefits, and the expected effect searched. Finally, all the favorable effects found with the diets have been improved when weight loss has been achieved, in the presence of overweight or obesity. The additional beneficial effects of each diet (on insulin sensitivity, micronutrient supply, antioxidant capacity, etc.) should be one of the keys to consider when prescribing a type of diet.

In this article, we have reviewed pieces of evidence on the effects of different diet types on NAFLD, and found that some of them exhibited good results on NAFLD, although some of them with limited evidence. Although, to our knowledge, only the Mediterranean Diet has been included in the main scientific societies’ reports on the management of NAFLD [20,21,22,23], evidence from observational and prospective studies suggests that the low-fat diet, the vegetarian diets, and some types of dietary interventions based on the time of eating, such as the intermittent diet, could be alternatives for treating NAFLD.

## Figures and Tables

**Table 2:** Summary of the main characteristics of ongoing trials investigating the effects of healthy diet on NAFLD. Abbreviations: NAFLD: non-alcoholic fatty liver disease; MRI: Magnetic Resonance; T2DM: type 2 diabetes.

Identifier	Study Design	Population Health Status	Sample Size	Duration	Type of Intervention	Type of Control	Liver Endpoint
NCT05309642	Parallel control trial	36 BMI >25 kg/m^2^ and 36 BMI < 25 kg/m^2^	72	12 weeks	Very low calories ketogenic diet	Standard of care diet	Change on liver steatosis by MRI
NCT05275608	Parallel control trial	BMI > 30–40 kg/m^2^ NAFLD T2DM	20	90 days	Very low calories ketogenic diet	Hypocaloric Mediterranean diet	Change in liver enzymes as secondary outcome
NCT04440540	Parallel control trial	Obese patients	40	1 year	Lifestyle program (diet not specified)	Standard of care diet	Change of intrahepatic triglycerides content in NAFLD
NCT05200585	Parallel	NAFLD	30	6 months	Behavioral: Healthy Liver/Hígado Sano program	Standard of care diet	MRI liver steatosis change
NCT04383951	Parallel	NAFLD	40	16 weeks	Ketogenic diet	Standard of care diet	MRI liver steatosis change
NCT05268042	Randomized controlled trial	Obese adolescents with NALF	80	6 months	Moderately carbohydrate-restricted diet	Fat-restricted control diet	MRI liver steatosis change
EudraCT:2021-000152-19	Randomized controlled trial	NAFLD and prediabetes patients	390	18 months	1.Med diet + metformin placebo pioglitazone placebo 2. Med diet + metformin + pioglitazone placebo 3. Med diet + metformin + pioglitazone	-	Evaluated witch model induce a greater regression of NAFLD

## Abbreviations

NAFLD	non-alcoholic fatty liver disease.
CVD	cardiovascular disease.
NO	nitric oxide.
MUFA	monounsaturated fatty acids.
n-3 PUFAs	omega 3 polyunsaturated fatty acids.
MRI	magnetic resonance imaging.
T2DM	type 2 diabetes.
